# CD19 CAR-T cell therapy for relapsed/refractory acute lymphoblastic leukemia: factors affecting toxicities and long-term efficacies

**DOI:** 10.1186/s13045-018-0593-5

**Published:** 2018-03-15

**Authors:** Li-Na Zhang, Yongping Song, Delong Liu

**Affiliations:** The affiliated Cancer Hospital of Zhengzhou University and Henan Cancer Hospital, 127 Dongming Road, Zhengzhou, 450008 China

**Keywords:** Chimeric antigen receptor, CAR-T, Cancer immunotherapy, Blinatumomab

## Abstract

The prognosis of adults with relapsed/refractory (R/R) acute lymphoblastic leukemia (ALL) remains dismal even at this day and age. With salvage chemotherapy, only 29% (range 18 to 44%) of the patients with R/R ALL can be induced into complete remission (CR), with a median overall survival (OS) of 4 months (range 2–6 months). Blinatumomab and inotuzumab ozogamycin (IO) are immunotherapeutic agents that increased CR to 80% and extended survival to 7.7 months in this high-risk population of patients. In the last few years, chimeric antigen receptor (CAR)––engineered T cells have led to major progress in cancer immunotherapy. CD-19 CAR-T cells have been recently approved for high-risk R/R ALL and lymphoma. The data from long-term follow-up of a single-center phase I study of 19-28z CAR-T cell therapy for adult R/R ALL were just published. At the same time, a multicenter phase II study of 19-41BB CAR-T cell therapy for children and young adults with R/R B cell ALL was also published. The two studies provided fresh information with long-term follow-up. This research highlight analyzed the data and proposed future perspectives for further investigation in this rapidly evolving field.

## Background

The prognosis of adults with relapsed/refractory (R/R) acute lymphoblastic leukemia (ALL) remains dismal even at this day and age [1, 2]. With salvage chemotherapy, only 29% (range 18 to 44%) of the patients with R/R ALL can be induced into complete remission (CR), with a median overall survival (OS) of 4 months (range 2–6 months) [3–10]. Novel agents are needed to improve the therapy of R/R ALL. Blinatumomab, the first FDA-approved BiTE antibody, has been shown to extend the median survival to 7.7 months in patients with CD19+, Philadelphia chromosome (Ph)––negative R/R ALL [11] (Table 1). Recently, inotuzumab ozogamycin (IO), a CD-22 antibody-drug conjugate, was shown to improve CR rate to 80% and overall survival to 7.7 months [12]. In the last few years, chimeric antigen receptor (CAR)––engineered T cells have led to major progress in cancer immunotherapy [13–25]. CD-19 CAR-T cells have been well studied and recently approved by FDA for children and young adults with R/R ALL (tisagenlecleucel, kymriah™) [24, 26–29]. In this population of patients, 90% of the patients were induced to CR, yet the median follow-up was only 7 months. A second CD-19 directed CAR-T cell product was recently approved for the therapy of relapsed and/or refractory lymphoma (axicabtagene ciloleucel, yescarta™) [30].

**Table 1:** New agents for immunotherapy of relapsed/refractory acute lymphoblastic leukemia

	Blin	IO	19-28Z CAR	19-41BB CAR
Phase	III	III	I	II
Patients				
Age (year)	≥ 18	≥ 18	≥ 18	≤ 21
No. enrolled	271	141	83	92
No. evaluable	267	109	53	75
Follow-up (m)	11.7	NA	29	13.1
CR %	44	80.7	83	81
EFS (m)	7.3	5 (PFS)	6.1	NR
OS (m)	7.7	7.7	12.9	NR
CRS (≥ grade III %)	4.9	NA	26	47
Neurotoxicity %	9.4	NA	44	40
References	11	12	31	32

Abbreviations: *Blin* blinatumomab, *IO* inotuzumab ozogamycin, *CAR* chimeric antigen receptor, *CRS* cytokine release syndrome, *m* month, *NA* not available/applicable, *NR* not reached, *PFS* progression-free survival, *CR* complete remission, *EFS* event-free survival, *OS* overall survival

## 19-28z CAR-T cells for R/R ALL in adults with a long-term follow-up

The data from a long-term follow-up of a single-center phase I study of 19-28z CAR-T cell therapy for adult R/R ALL were just published [31]. The primary endpoint of this phase I study was safety. This study had a median follow-up of 29 months (range 1–65 months), with 53 patients evaluable. The CR rate was 83%, and the median OS was 12.9 months. The rate of severe cytokine release syndrome (CRS) was 26% (Table 1). At the same time, a multicenter phase II study of 19-41BB CAR-T cell therapy for children and young adults with R/R B cell ALL was also published [32]. This study had 75 evaluable patients. The median follow-up in this study was 13.1 months. The CR rate was 81%. The rate of severe CRS was 77% [32, 33].

## The patient population

The 19-28Z CAR study enrolled a total of 83 patients over a stretch of 65 months, yet 30 patients did not receive the CAR-T therapy mainly due to the advanced disease status of the patients [31]. The 53 evaluable patients were indeed heavily pretreated: 23% with primary refractory disease, 61% had 3 or more lines of prior regimens, 36% failed prior allogeneic stem cell transplantation (alloSCT), and 25% had failed blinatumomab. Thirty percent (16 of 53) of the evaluable patients were Ph+, with 10 of 16 already failed ponatinib. Among the 53 patients, only 6 (11%) were negative for minimal residual disease (MRD) (< 0.01% bone marrow blasts) prior to CAR-T infusion.

## Factors affecting responses and toxicities

Although the 83% CR rate is not particularly impressive in the 19-28z study, 67% of the patients became MRD-negative [31]. It is noteworthy that the CR rate was not affected by any of the common poor-risk factors, indicating that CAR-T therapy may be able to defy conventional poor-risk factors (prior transplant history, prior regimens, conditioning regimens). Instead, disease burden and Ph positivity appeared to affect responses. Higher disease burden was associated with higher rate of CRS and neurotoxicity. Higher peak CAR-T cell expansion was noted to be associated with deeper responses (higher MRD-negative CR rate) but at the expense of higher rate of and more severe neurotoxicity and CRS. The study suggests that both higher disease burden and higher peak CAR-T cell expansion are independent predictors of severe neurotoxic effects.

In the 19-41BB CAR study, no relationship between peak CAR-T cell expansion and dosage of the infused CAR-T cells was observed. There was neither definitive relationship observed between dosage and response [32]. Severe CRS requiring ICU care was reported in 47% of the patients. This appeared to be higher than that observed in the 19-28z single-center study. However, the 19-41BB CAR study was an international multicenter study, and the criteria for ICU care varied widely and could potentially be responsible for the high rate of ICU care.

## Factors affecting relapses and long-term survival post-CAR-T therapy

In the 19-28z CAR study, 32 of the 53 patients (67%) had MRD-negative CR whereas 9 patients entered CR with still positive MRD (bone marrow blasts > = 0.01%) post CAR-T therapy. All patients with positive MRD CR (9/9) relapsed with CD-19+ blasts, but only 50% of those with MRD negative CR (16 of the 32) had a relapse, including 4 patients who had a relapse with CD19− blasts. Among the 32 patients with MRD-negative CR, allogeneic transplantation did not lead to better survival than those who were not transplanted. Although the sample size of this study was not powered to detect the potential differences, the above observation nevertheless implies that additional graft-versus-leukemia effect may not be achieved by alloSCT after CAR-T cell infusion, and additional factors are responsible for relapses, loss of CD19 expression being a prominent one. Expression of PD-L1 was reported to be increased in those patients who became refractory to blinatumomab [34]. It is not known at this time whether this could be one of the factors responsible for relapse. It is unclear at this time whether it is beneficial to use allogeneic CAR-T cells as conditioning therapy followed immediately by allogeneic stem cell transplantation [35].

The persistence of 19-28z CAR-T cells was not found to be correlated with survival in the study. This observation may lend support to the development of newer generation of CARs that have on-off switch to control the expression of the engineered CARs [16, 19, 20].

The 19-41BB CAR-T cells were demonstrated to be present in the peripheral blood for a median of 168 days, with ongoing persistence of 20 months. The 4-1BB costimulation domain has been suggested to be able to ameliorate T cell exhaustion and enhance the persistence of CAR-T cells. It remains unclear at this time whether prolonged persistence of the 19-41BB CAR-T cells could lead to better overall survival in the long run. In the current report with a 13.1 month median follow-up, both median event-free survival and overall survival have not been reached [32].

Neither CAR-T cell dose nor the magnitude of peak CAR-T cell expansion was associated with better survival, even though these are potential factors being associated with higher toxicities. It may be the ratio of the CART cell dose to the disease burden that is associated with better event-free and overall survival [31]. The median OS was 20.1 months among patients with low disease burden, as compared with 12.4 months among patients with a high-disease burden (*p* = 0.02). The study again confirmed the well-known phenomenon that lower disease burden is associated with better long-term survival in ALL. Great efforts should be made to achieve the lowest disease burden (MRD-negative CR) prior to CAR-T therapy as well as to alloSCT.

There have been a variety of studies using similar but different and/or independent CD19 CAR vectors with similar structures, even though these were targeting the similar type of cells [15, 16, 20, 26, 31, 36–45]. It is important to recognize that they should not be considered equal or interchangeable, neither should the outcomes from these studies be compared side by side since these were not randomized studies and there were multiple variables that were not directly comparable. For example, as shown in Table 1, there were differences in terms of EFS, OR, and CRS between the two CAR-T studies on R/R ALL, with the 19-41BB CAR-T having EFS and OS not reached. The EFS curve plateaus around 10 months in 19-41BB CAR trial, but there was no such plateau until 20 months on 19-28Z CAR-T trial. In addition, the 1 year EFS in 19-41BB CAR-T trial was 50%, whereas the EFS was about 20% in the 19-28Z CAR-T trial. It might seem that these results were quite different, though one should not directly compare them since the studies were not designed for the comparison, and there were multiple major differences, including patient populations, sample sizes, CAR constructs, and study center participations, as well as primary end points.

## Future perspectives

Antibodies such as blinatumomab and IO are proven to be superior than the conventional chemotherapeutic agents for the adult R/R ALL. CAR-T cells appear to be very promising in further improving the therapeutic benefits in this population with very poor prognosis. However, these are still early phase studies with relatively small sample sizes and short follow-ups. Future studies such as the following are clearly needed:

1. Large prospective studies must be done to confirm the clinical efficacy
  In the two large prospective randomized studies on adult R/R ALL, blinatumomab and IO were shown to extend median survival from under 6.7 to 7.7 months. Although it is not directly comparable, autologous CD-19-directed CAR-T cells were shown to lead to a median OS of 12.4 months in the 19-28z CAR study. For the phase II 19-41BB CAR study, the median overall survival has not been reached, and the median follow-up was only 13.1 months. As mentioned above, these results should not be compared directly. Large prospective studies must be done to confirm the clinical efficacy.
2. Moving CAR-T cell therapy to the front
  In the 19-28z CAR study, there was production failure of CAR-T cells in two subjects, and a total of 30 subjects failed to receive the CAR-T infusion, mostly due to advanced disease status. Therefore, it is conceivable that more patients could be treated with CAR-T cells if the therapy can be used earlier in the treatment planning.
3. Disease control should be optimized prior to CAR-T therapy
  Disease burden prior to CAR-T infusion was shown to be the single most important factor to predict favorable outcome. It is therefore critical to optimize disease control and eliminate MRD prior to CAR-T cell therapy. This further supports the notion that CAR-T cell therapy should be used earlier as a consolidation regimen since better disease control may be achieved earlier in the course of consolidation/salvage therapy. In addition, it is important to optimize the CAR-T cell dose so that better ratio of CAR-T cell dose to disease burden can be achieved.
4. Additional therapeutic modalities post CAR-T infusion should be explored
  All patients with MRD+ CR and 50% of those with MRD− CR relapsed after CAR-T cell therapy in the 19-28z CAR study. AlloSCT following CAR-T cell infusion in the patients with MRD− CR did not lead to better survival than those who were not transplanted. These intriguing observations suggest that therapeutic modalities other than AlloSCT post CAR-T cell therapy should be explored in order to achieve better long-term survival. Sequential and/or cocktail (concurrent) infusion of CAR-T cells targeting two or more different antigens are being explored [16, 46]. To reinvigorate CAR-T cells, epigenetic modulation, and PD-1 antibodies are also being investigated [47]. Allogeneic CAR-T cells, including haplo-identical CAR-T and universal CAR-T cells, are being studied in clinical trials [35, 48]. These may be considered as alternative options for those patients with high risk of relapses after autologous CAR-T cell therapy.

## Abbreviations

ALL: Acute lymphoblastic leukemia
AlloSCT: Allogeneic stem cell transplantation
CAR: Chimeric antigen receptor
CRS: Cytokine release syndrome
